# Deficient mechanosensation in *mec-3* decreases precipice response in *C. elegans*

**DOI:** 10.17912/micropub.biology.000429

**Published:** 2021-08-10

**Authors:** Robin M Mitchell, Diana S Pattillos, Shuyu Zhang, Jared J Young

**Affiliations:** 1 Biology Department, Mills College, Oakland, CA, 94613, USA

## Abstract

The precipice response in *Caenorhabditis elegans *is a little-understood phenomenon in which worms move rapidly away from edges. We hypothesized that mechanosensation underlies the precipice response and that mechanosensory mutants would exhibit the precipice response less often than N2 wild type worms. We found that *mec-3* mutants, with severe loss of mechanosensation, exhibited the precipice response at a lower rate than N2, but* mec-10 *and *trp-4* mutants, with partial loss of response to mechanical stimuli, responded at a similar rate to N2. These results provide a characterization of the precipice response and implicate a role for mechanosensation in this behavior.

**Figure 1. C. elegans frequently reverses at a cliff edge; response is reduced in mec-3 mutants:**
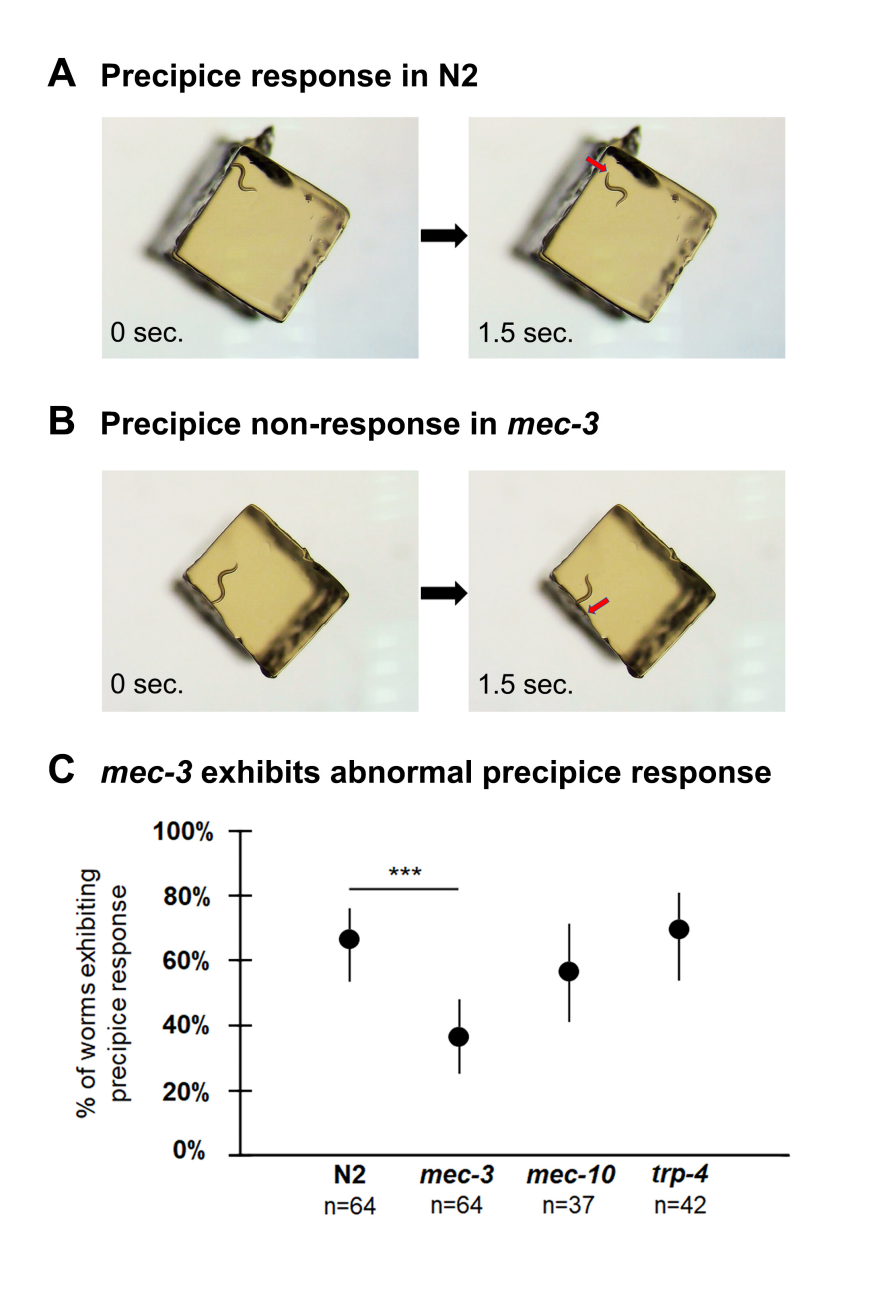
**A)** N2 worm exhibiting precipice response. The worm first encounters the edge of the agar chunk, with its nose going over the edge (left panel). The worm quickly reverses (direction indicated by red arrow) and is shown 1.5 seconds later (right panel). **B)** *mec-3* worm failing to exhibit precipice response. Upon its first encounter with the edge of the agar chunk (left panel), rather than reversing, this worm continues crawling down the side of the chunk (direction indicated by red arrow) and is shown 1.5 seconds later (right panel). **C)** Mutant worms with defects in mechanosensation were tested for their precipice response. *mec-3*, but neither *mec-10* nor *trp-4,* showed a significant decrease in the percentage of worms exhibiting the response. Observers in these experiments were blind to the genotype. Data in C represents % of worms exhibiting precipice response and 95% confidence interval. n = number of worms assayed. *** indicates p < 0.001, based on chi-square test, *X^2^* (1, *N*=128) = 11.28, p < 0.001. Chi-square test showed no significant difference in the likelihood of precipice response between *mec-10* and N2, *X^2^* (1, *N*=101) = .79, p = .38, or between *trp-4* and N2, *X^2^* (1, *N*=106) = 0.13, p = .71. The videos from which the still photos in A and B were taken are available in the Supplemental Materials. Magnification: 250X.

## Description

The roundworm *Caenorhabditis elegans* exhibits a variety of behaviors in response to external mechanical stimuli, such as reversing when prodded. One behavior that has remained uncharacterized until now is the *precipice response*, a phenomenon first alluded to by Chalfie *et al.* (2014) in which *C. elegans* move rapidly away from edges or gaps on an agar surface. Similar cliff avoidance behaviors have been observed in other species, such as mice, which sense cliffs using vision and their whiskers (Crawley 1999; Arakawa and Erzurumlu 2015). However, these sensory systems are lacking in *C. elegans*. We hypothesized that the precipice response is mediated at least in part by mechanosensation, as touch receptor and/or proprioceptive neurons might respond when there is no longer a solid surface supporting the worm from underneath. If this hypothesis is correct, we might observe a reduced precipice response in mechanosensory mutants. Our experiments sought to 1) characterize the precipice response and 2) explore the role of mechanosensation in this behavior.

To characterize the precipice response, we first observed N2 wild type worms crawling on small (2 mm^2^) agar chunks, noting their response to encountering the edge of the chunk. In most cases, as soon as the worm’s nose went over the edge, the animal would strongly reverse (Figure 1A). We settled on a definition of the precipice response which captures its essential features: within 2 seconds of the nose moving off the edge of the agar chunk, the worm begins a reversal that completes at least one full sine wave. We only scored the precipice response the first time that a worm encountered the edge in order to avoid any adaptation effects.

To investigate the contribution of mechanosensation to the precipice response, we used three mutants with distinct defects. *mec-3* encodes a homeobox transcription factor required for the differentiation of all six touch receptor neurons (Way and Chalfie 1988), and *mec-3* mutants do not respond to any mechanical stimuli (Chalfie and Sulston 1981). *mec-10* encodes a component of the DEG/ENaC touch transduction channel complex. *mec-10* mutants show defects in gentle but not harsh touch, with the *e1515* allele showing roughly 70% reduction in both gentle touch responses and mechanoreceptor currents (Árnadóttir 2011). *trp-4* encodes a subunit of a TRP mechanosensory channel (Kang 2010). *trp-4* mutants show neural activity in response to a nose press, but not a gentler nose buzz (Kindt 2007), and also show defects in proprioception (Li 2006), posterior harsh touch (Li 2011), and movement in 3D environments (Kwon 2015, Han 2017).

To confirm reported mechanosensory defects in these mutants, we performed light and harsh touch assays. *mec-3* was completely unresponsive to both (0 out of 10 responded to light touch, 0 out of 10 responded to harsh touch). *mec-10* showed a normal response to harsh touch (10 out of 10 responded), and a strong reduction in gentle touch response similar to previous reports (2 out of 10 responded, compared to 10 out of 10 for N2). *trp-4* showed normal responses to both light and harsh touch (10 out of 10 responded to light touch, 10 out of 10 responded to harsh touch). For *trp-4*, we also performed nose touch assays (Chalfie 2014) and found *trp-4* to exhibit normal responses (10 out of 10 responded). We speculate that we failed to observe a defect in behavior of *trp-4* in these assays because the nose touch assay produces a stimulus that is more similar to a nose press than the gentler nose buzz.

We then tested these three mutant genotypes in the precipice response assay. Of these mutants, only *mec-3* showed a significantly lower precipice response frequency than N2 (Figure 1B,C). This shows that mechanosensation is indeed involved in the precipice response. Our results furthermore suggest that a mechanical stimulus related to harsh touch on the anterior end may be involved in triggering the response, since that system is impaired in *mec-3,* but not in *mec-10* or *trp-4*.

One confound in our experiments is that, as we defined it, the precipice response requires the worm to execute a strong reversal of at least one sine wave. Thus, it is possible that a mutant may be scored as executing fewer precipice responses not due to its response to the edge per se, but due to a general defect in executing strong reversals. It is the case that both *mec-10* and *trp-4* have reported locomotory defects (Li 2006; Yemini 2013). However, these defects did not appear to impair the ability of these mutants to execute the precipice response, as their response frequency was similar to that of the wild type. *mec-3* is quite sluggish but is still capable of executing precipice responses. *mec-3* also reverses spontaneously at the same rate as N2, suggesting that *mec-3* does not have difficulty in initiating reversals (Zhao *et al.* 2003). Nevertheless, it may be that the *mec-3* phenotype is at least partly due to a general defect in locomotion, and is not solely due to a defect in its response to the edge.

We speculate that the direct stimulus that triggers the precipice response is either the sudden sensation of a lack of a solid substrate under the head, or the sensation of gravity pulling the head down when the head is no longer supported by the substrate, or both. By reversing, the worm avoids moving quickly over an edge in a way that may result in injury. The experience of having its head move suddenly off the substrate is likely to be fairly novel for a worm reared on an agar plate. It would be interesting to see if the response differs in worms grown in more varied 3D environments (e.g. Kwon 2015, Guisnet 2021). *C. elegans* behavioral responses to gravity are uncharacterized, and possibly nonexistent (Okumura *et al.* 2013). However, physiological responses to gravity have been documented, and some of these require mechanosensation (Kim *et al.* 2007). This study provides a characterization of the precipice response in *C. elegans* and serves as a first step in discerning its mechanisms. Further research is necessary to understand the broader role of the precipice response in behavior, and to gain a deeper appreciation of mechanosensation’s role in this response.

## Methods

***Animal maintenance***

*C. elegans* maintenance was carried out using standard techniques (Brenner 1974). Worms were grown at 19˚C on 6 cm Nematode Growth Medium (NGM) plates seeded with the OP50 strain of *E. coli*. All worm strains were obtained from the *C. elegans* Genetics Center (CGC): *mec-3(e1338), mec-10(e1515), trp-4(sy695).* Wild type strain used was the Bristol strain N2.

***Precipice assays***

Young adult hermaphrodites were used for precipice assays. To perform the assay, a chunk of unseeded NGM agar with a surface area of approximately 2 mm^2^ was cut from an NGM plate and one or two worms were transferred to it using a platinum wire pick or dog whisker pick. Immediately after placing the worm on the agar surface, the worm was placed on a stereomicroscope and its behavior recorded on video under 120x or 250x magnification. The reaction of each worm upon first encountering the edge of the agar surface was scored by watching the videos. Each worm was scored by two independent observers who were blind to the worm’s genotype. In the event that the two observers’ calls for a given worm did not agree, four researchers (all blinded to the genotype) watched the video of the worm together and came to a consensus on the call.

***Touch assays***

Young adult hermaphrodites were used for touch assays. Gentle touch to the head was performed as described (Chalfie 2014) using an eyelash pick. Harsh touch to the midsection was performed as described (Way 1989) using a platinum wire pick. Nose touch was performed as described (Chalfie 2014) using an eyelash pick.
